# Early discontinuation of long-acting reversible contraceptives and associated factors among women discontinuing long-acting reversible contraceptives at national referral hospital, Kampala-Uganda; a cross-sectional study

**DOI:** 10.1186/s40834-023-00223-1

**Published:** 2023-04-12

**Authors:** Agery Bameka, Othman Kakaire, Dan Kabonge Kaye, Fatuma Namusoke

**Affiliations:** Department of Obstetrics and Gynecology, College of Health Sciences, Makerere University, Kampala, Uganda

**Keywords:** First-year discontinuation, Long-acting reversible contraceptive, Subdermal implant, Intrauterine contraceptive devices

## Abstract

**Background:**

High levels of unmet need for contraception and unwanted pregnancies are high in developing countries despite efforts to reduce them. Long-acting reversible contraceptive (LARC) methods are more than 99% effective in preventing pregnancy. Discontinuation of LARC within the first year of initiation contributes to the high levels of unmet need. This study aimed to determine the prevalence and factors associated with the first-year discontinuation of LARC at Kawempe National Referral hospital.

**Methods:**

A facility-based cross-sectional study was conducted from February 2020 to June 2021. We consecutively recruited 354 participants who discontinued a LARC (intrauterine device {IUD} and sub-dermal implant) during the study period after informed written consent. Data on duration of use, reasons for discontinuation, and factors associated were collected using a face-to-face interviewer-administered questionnaire and review of client records. Early LARC discontinuation was defined as the termination of the contraception within the first 12 months of use. Data were entered using SPSS version 14/0 and analyzed in STATA version 15. Prevalence was expressed as a proportion while logistic regression was used to assess factors associated with early LARC discontinuation. Variables with a p-value of < 0.05 were considered statistically significant.

**Results:**

The proportion of first-year discontinuation of LARC was 29%. Women Age less than 25 years (OR = 5.07; 95% CI: 1.1–24.8) and those who desired a family size of fewer than four children (OR = 3.19; 95%CI: 1.2–8.7 ) were more likely to discontinue the LARC within 12 months of initiation after multivariate analysis. Method-related **r**easons for removal were painful menstrual cramps for implants, recurrent infections for IUDs, and a non-side effect reason was the desire to get pregnant.

**Conclusion:**

A high proportion of women discontinue LARC within 12 months following initiation. Young adults and those who desire small families are more likely to have first-year discontinuation of LARC. We recommend age-specific counseling for patients receiving the LARC and further studies looking at the depth analysis of reasons for the first-year discontinuation.

**Supplementary Information:**

The online version contains supplementary material available at 10.1186/s40834-023-00223-1.

## Background

In 2017, it was estimated that 800 women died daily as a result of pregnancy and related complications, and yet with each death 20 suffer serious morbidities associated with pregnancy globally [1]. Its estimated that  68% of global maternal deaths occur in sub-Saharan Africa. The lifetime risk of maternal death is higher in countries with high fertility rates. Reducing or limiting fertility is one of the interventions that can help to reduce maternal mortality.

More maternal deaths coupled with fertility rates contribute to disparities in the lifetime risk of maternal death which is 1 in 45 in low-income countries, compared to 1 in 5,400 in high-income countries. Women in sub-Saharan Africa face the highest lifetime risk (1 in 38), followed by South Asia (1 in 240) globally. Modern contraceptive use has been linked directly to fertility which is a key factor in the lifetime risk of maternal death [2].

Uganda has a high fertility rate despite improvement in the uptake of modern contraceptives [3]. An increase in contraceptive prevalence rates has been linked to a reduction in maternal and perinatal mortality and morbidity [2].This is achieved by reducing unwanted pregnancies which are linked to a reduction in unsafe abortions, the number of pregnancies that are at risk, and the total number of pregnancies [1].

Modern contraceptive use offers intervention for preventing unwanted pregnancy. This however is met with another challenge the high unmet need for contraception. In Uganda, 30% of women have an unmet need for contraceptive use [3]. The use of long-acting reversible contraceptives (LARC) is highly effective in preventing unwanted pregnancy. The cost-effectiveness of LARC is however affected by early discontinuation [4]. Contraceptive discontinuation in clients who have no desire for pregnancy indicate a missed opportunity to promote and sustain contraceptive use, as it leads to a high unmet need for family planning (FP) [5]. It presents a unique challenge in low and middle-income countries where funds for medical care are limited.

Long-acting reversible contraceptives are effective with a contraceptive failure rate of less than 1% [6]. They provide the most effective, efficient, and satisfying mode of contraception in resource-limited settings and hard-to-reach areas [7]. Modern contraceptive continuation rates vary widely globally for various reasons [5, 6].

 According to 2016 Uganda Demographic Health Survey (UDHS), all methods modern contraceptives discontinuation within 12 months was 45% with more than a third discontinuing because of the side effect. There is limited data on rates and what drives the first-year discontinuation of LARC. The objective of this study was to determine the prevalence of first-year discontinuation of LARC and associated factors at Kawempe National Referral Hospital.

## Methods

### Study design

A facility-based cross-sectional study among women seeking care for discontinuation of a LARC at Kawempe National Referral Hospital.

### Study setting

The study was conducted at Kawempe National Referral Hospital. The hospital is located in the Kawempe Division, one of the five administrative units of the Kampala Capital City Authority. This hospital is located approximately 5 km by road, north of the city’s central business district, along the Kampala-Gulu Highway. The hospital is a National Referral Hospital and a teaching hospital for Makerere University College of Sciences offering maternal and neonatal health services. The FP clinic runs from Monday to Friday, 0800 to 1700 hours with five midwives on duty. It is a midwife-led clinic but gynecologists are available for consultation. The clinic offers services including Contraception, Cancer of cervix screening, HIV counseling, and testing services, and follow-up of clients with contraceptive-related side effects. The clients receive group and individual counseling sessions with a midwife trained in family planning before the insertion and discontinuation of a method. The study was approved by the Makerere University School of Medicine Research and Ethics Committee (#REC REF 2020 − 163.)

### Study population

All women who discontinued a LARC method at Kawempe NRH during the study period. They were approached at the exit from the family planning clinic and recruited after informed written consent.

### Data collection

Data were collected using an interviewer-administered questionnaire that included open-end and closed-end questions on socio-demographic (age, marital status, religion, occupation, and education), obstetric (number of children, counseling, past contraception utilization), method-related (side effects, follow-up, conception, past contraception utilization, and social (partner opposition and involvement, peer influence) factors. Age in completed years and duration of use of LARC was collected as a continiuos variable.

### Sample size determination

The sample size for objective one looking at the prevalence of first-year discontinuation of LARC was determined using the Kish, *Leslie* 1965 formula. n = Z^2^P (1-P)/d^2^, n = desired sample size, z = confidence interval.

Z = 1.96, P = 0.45, Delta (d) = 0.05; gave a population of 204 women. P is the proportion of women that had early discontinuation of Implanon [8].

The sample size for objective two for determining factors associated with the first-year discontinuation of LARC was calculated using the Fleiss formula [9]. Using results from the study by Tsirity and others in Ethiopia, using counseling before insertion with early discontinuation of Implanon [8]. The standard normal value corresponding to the 5% level of significance and 80% power of the study gave a sample size of 349 participants. Since 349 participants were the larger sample size from the calculations and it was used for the study. Five participants were added to the calculated sample size to account for non-respondents. Therefore, the sample size was 354 mothers.

### Sampling procedure

Women were recruited consecutively until the sample size was realized. We approached them at the exit area of the family planning clinic and sought consent after thorough explanations about the study.

### Inclusion criteria

Women of reproductive age (15–49 years) who had discontinued a LARC.

### Exclusion criteria

Women who were too sick to respond to the questionnaire and those who had no record of the date of LARC insertion and could not remember the month and year of insertion were excluded from the study.

### Data collection instruments and definition of variables

Data were collected using a semi-structured interviewer-administered questionnaire. Data on the socio-demographic, Obstetric, and method-related characteristics. All participants were interviewed on the day they discontinued the LARC method. Data on duration/date of initiation were collected using client card/ self-reported history and, for those that had no exact date of initiation, we got the month of insertion and the 15th day was taken as the date of insertion.

**Dependent variable**; First-year LARC discontinuation was the dependent variable, defined as discontinuation at 12 months or less after LARC insertion. Long-acting reversible contraceptives in this study were subdermal implants and IUDs. It was measured as a binary variable coded as ‘Yes’ if a LARC was discontinued within 12 months of insertion and ‘No’ if 12 or more months.

**Independent variables**; included; socio-demographic factors (age, marital status, religion, occupation, and education), obstetric factors (number of children and number of induced abortions), social factors (husband perception and peer group influence), and method-related factors (type and duration of usage, side effects, follow up, conception, counseling, past contraception utilization).

### Data collection procedure

The data collection tool was developed after adapting questions from women and sociodemographic questionnaires [3] with some modifications. Data on the sociodemographic, obstetric characteristics, and method-related and social factors were collected using an interviewer-administered questionnaire. Data on duration/date of initiation was collected using client card/ self-reported history and for those that had no exact date of initiation, we got the month of insertion and the 15th day was taken as the date of insertion.

Participants were recruited at the family planning clinic, Kawempe NRH, every Monday to Friday, between 0800 and 1600 h. Data were collected from February 2020 to June 2021 using face-to-face interviews. The participants were recruited after written informed consent. Data were collected by four female research assistants, who were trained on the protocol, and study instruments before the initiation of the study. The research assistants were midwives working in the family planning clinic.

### Data management

The filled questionnaires were entered using Statistical Package for Social Services (SPSS) version 14/0 and later exported to STATA version 15. Data were cleaned, coded, and analyzed in STATA version 15. To ensure the confidentiality of the respondents’ details, the questionnaires were kept away in a secure place after data entry and only accessed by the principal investigator. Names of the respondents were not written on the questionnaires, we used serial numbers for identification.

### Data analysis

Descriptive statistics including proportion, percentage, mean, median, and standard deviation were presented in text and tables. The prevalence was expressed as a proportion with the denominator being all women discontinuing LARC and the numerator being women who discontinued within 12 months following initiation. The reasons for the discontinuation of LARC were summarized in frequencies. The factors associated with first-year discontinuation were assessed using logistic regression analysis. Variables with a p-value of 0.20 or less at bivariate analysis were included in the multivariate model to determine factors associated with the first-year discontinuation of LARC. Age of partcipants in years and desired number of children were included in the model as continous variables. The significance level was set at 5% for all tests of significance.

## Results

### Baseline characteristics of the study participants

Socio-demographic characteristics of the study participants.

A total of 354 participants were enrolled between February and June 2021. The majority of the participants were married (80%) and came from urban areas (92%). Almost half (48%) of women had attained secondary education and 46% of the husbands of the women recruited in the study attained secondary education. Details of other demographic characteristics of participants are shown in Table 1.

**Table 1:** Demographic characteristics of the participants

	Frequency(n = 354)	Percentages (%)
**Age of the participant**		
**Less than 25 years**	89	25
**25–29 years**	115	32
**30–34 years**	74	21
**35–39 years**	43	12
**>=40 years**	33	9
**Marital status**		
**Married**	284	80
**Not married**	70	20
**Religion**		
**Anglican**	119	34
**Catholic**	115	33
**Moslem**	71	20
**Other**	48	14
**Residence**		
**Urban**	325	92
**Rural**	29	8
**Level of education of woman**		
**Illiterate/primary**	105	30
**Secondary**	170	48
**College and beyond**	79	22
**Level of education of husband**		
**Illiterate/primary**	53	18
**Secondary**	138	46
**College and beyond**	111	37
**Monthly income of a woman**		
**<=1,000,000**	272	84
**> 1000,000**	51	16
**Monthly income of the husband**		
**<=1,000,000**	235	78
**> 1000,000**	68	22

### Obstetric factors for study participants

74% had less than four living children, 70% had no history of abortion, and 49% wanted to conceive within one year. More than half 53% had a history of contraceptive use before the LARC of whom 48% had used injectable contraceptives. Counseling before initiating the discontinued LARC was offered to 89% of the participants, almost all 99% made their own choice to have the LARC method initiated and 69% were followed up after method initiation. Details of the obstetric factors in the study participants are shown in Table 2.

**Table 2:** Obstetric history of study participants

	Frequency(n = 354)	Percentages (%)
Number of live children		
0	16	5
< 4	263	74
>=4	75	21
History of abortion		
Yes	105	30
No	249	70
Desired number of children		
< 4	65	21
>=4	251	79
When do you plan to get pregnant		
Not at all	51	15
Within one year	164	49
For more than a year	122	36
History of ever use of contraception		
Yes	189	53
No	165	47
Family planning method ever used		
OCP	47	19
Injectable	121	48
OCP & Injectable	17	7
Others	68	27
Where LARC was inserted		
Govt health facility	316	89
Private health facility	38	11
Counselling offered		
Yes	345	97
No	9	3
Who took the decision to use LARC		
Own choice	349	99
Health professional	5	1
Followed after insertion		
Yes	243	69
No	111	31
LARC Discontinued	354	100
Subdermal Implants	192	54.2
IUD	162	45.8

### Discontinuation of long-acting reversible contraceptives

The prevalence of first-year discontinuation of LARC among study participants was 29%. The average time to discontinuation for both IUD and subdermal implants was 24 (Standard deviation [ SD] 21.0) months. Method-specific time to discontinuation was 28.3 s (SD 26.9) and 20.3 (SD13.3) months for IUD and implants respectively.

About half of the participants 50.6% discontinued because of a desire for pregnancy. The method-related reasons for contraceptive discontinuation were menstrual problems for subdermal implants and recurrent infections from IUDs. Details of the reasons for discontinuation are shown in Table 3.

**Table 3:** Reasons for discontinuation of long-acting reversible contraceptives

LARC Method	Reason for discontinuation	Number	Percentage
IUD	Expired	4	(7.55)
	Thread irritates husband	6	(3.77)
	Recurrent infections	13	(24.53)
	Others	30	(56.60)
IMPLANT	Expired	15	(18.29)
	Weight gain	5	(6.10)
	Unusual headache	11	(13.41)
	Insertion arm pain	1	(1.22)
	Menstrual pain	50	(60.98)
Non-method-related reasons	Desire for pregnancy	127	(50.60)
	Husband rejection	10	(3.98)
	Divorced	7	(2.79)

Pie Chart showing first-year discontinuation of LARC.


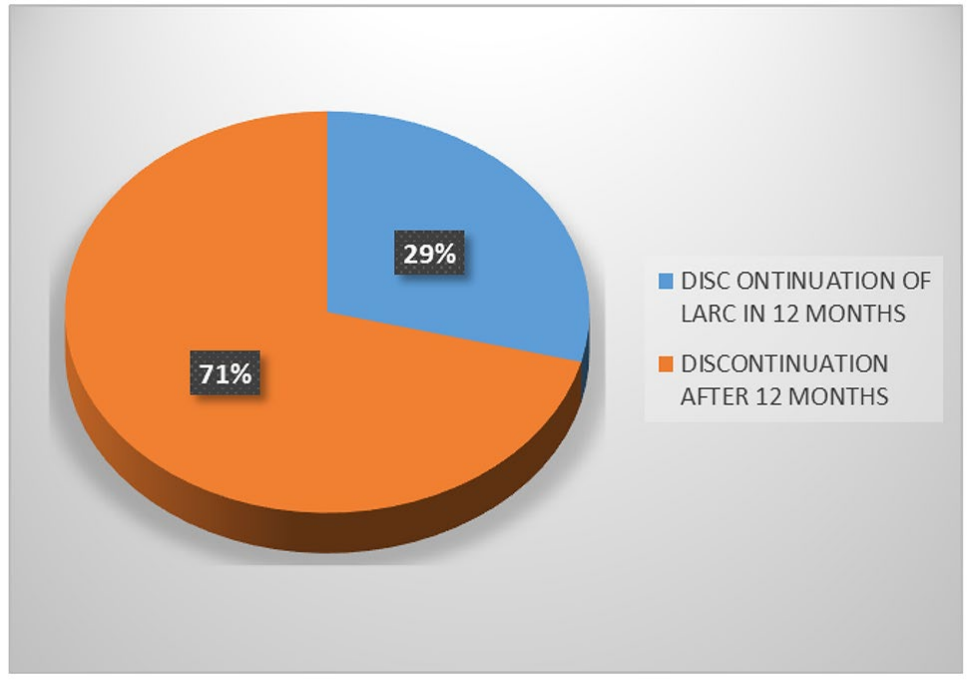


### Factors associated with first-year discontinuation of LARC

The older participants (aOR 0.925, 95%CI [0.8–0.9]), and those who desired more children ( aOR 0.799, 95%CI [0.6–0.9]) were less likely to discontinue the LARC within 12 months. Compared to those who wanted pregnancy in more than a year, participants who did not want it at all were less likely to have first-year discontinuation (aOR 2.407, 95%CI [0.9–6.4]. Details of factors associated with first-year LARC discontinuation are shown in Table 4.

**Table 4:** Factors associated with first-year discontinuation of LARC among women using LARC at Kawempe National Referral Hospital (N = 354)

	**COR	95%C.I	P-Value	*** aOR	95% C.I	P-Value
Age of the participant	0.958	0.92–0.99	0.024*	**0.925**	**0.8–0.9**	0.005*
Desired number of children	0.619	0.3–1.1	0.102	**0.799**	**0.6–0.9**	0.044*
Education level of the Husband						
Illiterate/primary	1.797	0.9–3.6	0.102	1.345	0.6–3.2	0.502
Secondary	1.126	0.6-2.0	0.681	1.255	0.7–2.4	0.493
College and beyond	1			1		
Monthly income of husband UGX)^1^						
<=1,000,000	1.656	0.9–3.1	0.12	0.910	0.5–1.8	0.792
> 1000,000	1			1		
When do you plan to get pregnant						
Not at all	1.313	0.7–2.6	0.436	2.407	**0.9–6.4**	**0.08**
Within one year	0.690	0.4–1.7	0.166	0.744	0.4–1.4	0.354
More than a year	1			1		
Who took the decision to use LARC						
own choice	1			1		
Health professional	3.842	0.6–23.3	0.144	4.471	0.4–45.7	0.207

*p < 0.05: significant variables; **COR: crude odds ratios ***aOR: Adjusted odds ratio ^1^ Uganda Shillings

## Discussion

In this study, 29% of the participants discontinued the LARC within 12 months. with mean of 24 ± 21months. The young mothers and those who desired fewer children were more likely to discontinue a LARC within the first year of initiation.

Almost half of the participants discontinued the LARC due to desire for pregnancy. Discontinuing contraception with no pregnancy desire has been linked to high rates of unwanted and unplanned pregnancies [10]. Method-related reasons have been reported as causes of contraceptive discontinuation across the region [11] as reported in our study. Discontinuing contraceptives due to method-related reasons reflects the quality of counseling and after-service care given to clients at the family planning service centers [12]. Menstrual abnormalities were the leading cause of method-related discontinuation in participants using Implanon as reported previously [13].

The proportion of participants who discontinued a LARC within the first year in this study is lower than that due to sub-dermal implants alone (42%) in the same setting [14]. This is can be explained by the high side effect profile of subdermal implants compared to IUDs IUD resulting in less user satisfaction. The high first-year LARC discontinuation rates may be linked to the quality of counseling services. Poor quality of contraceptive counseling has been associated with contraceptive discontinuation [15]. The burden of first-year discontinuation was much higher than 13.5% among Implanon users in Egypt and USA [20, 16] and 8.1% in Nigeria [17]. In Ethiopia however, the prevalence of discontinuation of LARC within one year was 69.8% [18]. The differences in these may be a reflection of the quality of care in the different regions/countries.

The young participants were more likely to discontinue the LARC within 12 months following initiation than their counterparts. This is consistent with previous studies showing higher odds of discontinuing long and short-acting contraceptives among young people [19, 21–23]. Adolescents and young people are likely to be affected by negative peer influence which is associated with contraceptive discontinuation [24]. Giving client-centered counseling in adolescents has been suggested as a intervention to reduce contraceptive discontinuation [25]. Adolescents’ concern about future fertility [23] could explain the increase in first-year discontinuation rates among young poeple. Using IUD has however not been associated with reduction in fertility [29].

Participants who desired a small family size were more likely to discontinue the LARC within the first year. Those who desire a small family size are likely to have low parity yet it has been described as an independent risk factor for early contraceptive discontinuation [26, 27]. The high discontinuation rates in those who desire a small family size, may be explained by the perceived fear of infertility following contraceptives use as has been reported in a study in Uganda [28]. Further evaluation on contraceptive continuation among women of low parity is recommended.

### Study limitations

It was a hospital-based study that was prone to social desirability bias, especially concerning the reason for discontinuation. This was minimized by the recruitment of participants as they exited the facility. The cross-sectional nature of the study did not allow for looking at some clients who discontinued early from other facilities which could lead to an underestimation of the first-year discontinuation rates in this setting. Odds ratio used for assessing association may have overestimated the risk because the prevalance of first year discontinuation was more than 10%. 

About a third of women discontinuing a long-acting reversible contraceptive are doing it within the first year of initiation which undermines their cost effectiveness in this setting. First-year discontinuation of LARC is more likely in the young and those who desire a small family size. 

The results of this study looked at the first-year discontinuation of LARC at Kawempe National Referral hospital, an urban public health facility offering free services. The results from this study are generalizable to population in similar settings.

### Recommendations

Appropriate counseling and follow-up of younger people on LARC are necessary to capture early removal and side effects-related complaints. Counseling should be age specific as different age groups tend to have different concerns.Women desiring small families should be actively supported while using LARC. A study looking at fertility desires among people discontinuing contraceptives in this setting is recommended.Using exploratory study designs to determine why adolescents discontinue LARC is recommended.

### Electronic supplementary material

Below is the link to the electronic supplementary material.

Supplementary Material 1

## Data Availability

The datasets used and analyzed during the current study are available from the corresponding author upon reasonable request.

## List of abbreviations

IUD: Intrauterine Contraceptive Device
LARC: Long-acting reversible contraception
NGO: Non-Government Organization
SDGs: Sustainable Development Goals
SPSS: Statistical Package for Social Science
STI: Sexually transmitted infection
TFR: Total fertility rate
UDHS: Uganda Demographic and Health Survey
KNRH: Kawempe National Referral Hospital
