# Correction: Early discontinuation of long-acting reversible contraceptives and associated factors among women discontinuing long-acting reversible contraceptives at national referral hospital, Kampala-Uganda; a cross-sectional study

**DOI:** 10.1186/s40834-023-00250-y

**Published:** 2023-10-18

**Authors:** Agery Bameka, Othman Kakaire, Dan Kabonge Kaye, Fatuma Namusoke

**Affiliations:** Department of Obstetrics and Gynecology, College of Health Sciences, Makerere University, Kampala, Uganda

**Correction: Contracept Reprod Med 8, 27 (2023).**

10.1186/s40834-023-00223-1

Following publication of the original article [1], the authors regret Research reported in this publication was supported by the Fogarty International Center of the National Institutes of Health, U.S. Department of State’s Office of the U.S. Global AIDS Coordinator and Health Diplomacy (S/GAC), and President’s Emergency Plan for AIDS Relief (PEPFAR) under Award Number 1R25TW011213. The content is solely the responsibility of the authors and does not necessarily represent the official views of the National Institutes of Health. It should be added in the acknowledgment section.

The authors would like to apologise for any inconvenience caused.

The original article [1] has been updated.
