# Supplementary material for: Early discontinuation of long-acting reversible contraceptives and associated factors among women discontinuing long-acting reversible contraceptives at national referral hospital, Kampala-Uganda; a cross-sectional study
Source: Contracept Reprod Med. 2023 Apr 12;8:27. doi: 10.1186/s40834-023-00223-1 (PMC10091590; doi:10.1186/s40834-023-00223-1)
Supplement: Supplementary file 1 — Supplementary Material 1 [file 40834_2023_223_MOESM1_ESM.docx]

**APPENDIX 1; QUESTIONNAIRE**

Prevalence and factors associated with early discontinuation of Long-Acting Reversible Contraceptives among women using Long-acting reversible contraceptives at KNRH.

Date …………………….

Study number and Initials ………………………..

## Social demographic data

1. Woman’s age (completed years)

………

2. Are you married? ( Tick appropriately)

1. Married
2. Single
3. Divorced
4. Widowed

3. What is your Religion?

1. Anglican
2. Catholic
3. Moslem
4. Others specify

4. Where do you stay?

1. Urban
2. Rural

5.Highest level of education attained by the participant

1. Illiterate
2. Primary
3. Secondary
4. College and above

6. Highest level of education attained by the husband/partner

1. Illiterate
2. Primary
3. Secondary
4. College and above
5. Don’t Know

7. On average, how much money do you earn monthly?

1. <100,000 Ugx
2. >100,000 <1000, 000 Ugx
3. >1000, 000 Ugx

8. On average, how much does your husband/partner earn monthly?

1. <100,000 Ugx
2. >100,000 <1000, 000 Ugx
3. >1000,000 Ugx
4. Don’t Know

## Obstetric factors

9. How many children at or more than 7 months have you delivered?

………..

10. How many living children do you have?

…………

11. Have you ever lost a pregnancy?

1. Yes
2. No

12. How many children do you plan to have in life?

  ……….

13. When do you want become pregnant? ( in months)

  …………

## Contraceptive related characteristics

14. Did you use any other modern contraception method before Implanon or IUD?

1. Yes
2. No

15. If yes, which modern family planning method did you use before Implanon or IUD

1. OCP
2. Injectable
3. OCP and injectable
4. Others specify………………………

16. When was the LARC inserted?

……….

17. Where was the LARC inserted from?

1. Government health Centre
2. Private Health Centre

18. Were there counselling services offered before insertion of LARC method?

1. Yes
2. No

19. Who took the decision to choose the LARC method?

1. Own choice
2. Health professional
3. Others specify…..

20. Were you followed up after insertion?

1. Yes
2. No

21. Why have you removed the LARC?

**IUD**

1. Expired
2. Threads irritate husband
3. Irritates the man during intercourse
4. Recurrent infections
5. Others specify……...

22. Why have you removed the LARC?

**Implants**

1. Expired
2. Weight gain
3. Unusual headache
4. Insertion arm pain
5. Menstrual disruption
6. Others specify

23. For Both IUD and implants ; Non-side effect removal reasons

1. Desire for pregnancy
2. Husband objection
3. Divorced
4. Husband went abroad
5. Others specify………….

END

Thank you very much

Initials of person administering the questionnaire……………….
